# The Effect of Irrigation-Initiation Timing on the Phenolic Composition and Overall Quality of Cabernet Sauvignon Wines Grown in a Semi-Arid Climate

**DOI:** 10.3390/foods11050770

**Published:** 2022-03-07

**Authors:** Elyashiv Drori, Sarel Munitz, Ania Pinkus, Maria Stanevsky, Yishai Netzer

**Affiliations:** 1 Chemical Engineering Department, Ariel University, Ariel 40700, Israel; droris@ariel.ac.il (E.D.); anyutik81@gmail.com (A.P.); 2 Eastern Regional R&D Center, Ariel 40700, Israel; sarel.munitz@carmelwines.co.il (S.M.); mariar@ariel.ac.il (M.S.); 3 Carmel Winery, Soham 608500, Israel

**Keywords:** *Vitis vinifera*, red wine quality, irrigation, drought stress, phenols, organoleptic taste

## Abstract

In semi-arid areas, vineyards grown for winemaking are usually mildly irrigated by drip irrigation systems in a manner maintaining drought stress. This practice ensures the proper development of vegetative and reproductive organs on the one hand, and on the other, the development of high-quality grapes which can be hampered by overly abundant water application. In previous work, we have developed and demonstrated an irrigation model suitable for high-quality grape production in semi-arid areas. Here, we tackle the question of proper irrigation initiation dates—should one wait for vines to develop drought stress before the initiation of irrigation, or rather commence irrigation earlier? Our results show that vines which undergo initial irrigation late in the growing season tend to develop a lower midday stem water potential even after irrigation initiation. In addition, these vines tend to produce a lower number of bunches per vine and smaller berry size, leading to lower yields. The wine produced from the late-irrigated treatments had a higher phenolic content, primarily due to higher levels of catechin and epicatechin. Their levels increased as irrigation initiation dates were delayed, while caffeic acid levels showed an opposite trend. Late irrigation also led to higher color intensities compared to those of irrigation at earlier stages, due to higher levels of most anthocyanins. Finally, we show that the overall wine sensory score, representing its overall quality, was approximately five points higher for wines made from delayed irrigation treatments compared to wines made from early season irrigation treatments.

## 1. Introduction

Grapevine (*Vitis vinifera*) is one of the most widely grown fruits globally, primarily for wine production. Over recent years, the global warming phenomenon has been causing temperature increases during ripening time, which have led to wine quality and varietal typicity changes in many growing regions [1]. Using the currently available data, different models predict a likely temperature rise between 1.8 and 4.0 °C by the year 2100 [2]. This temperature increase might significantly reduce the areas suitable for grapevine cultivation for wine production in the coming years [3]. Other critical agricultural consequences of climate change may include an increase in the intensity of drought due to the augmentation of the evaporative atmospheric demand resulting from warming, which is expected to lead to altered water availability to the vines [4,5].

In the cultivation of vines for wine production, deficit irrigation is a common and necessary practice in order to achieve mild drought stress, which in turn will enhance wine quality [6,7,8,9,10,11]. In semi-arid regions such as Israel, one of the common viticulture practices for producing high-quality wines is to apply a deficit irrigation regime during the late stages of berry development. This practice is based on the fact that regulated drought stress during late stages of berry development (stages II and III) increases sugar accumulation, color, and aroma intensity in berries [8,9,12,13,14,15]. The severity of the deficit irrigation imposed on the vineyard depends considerably on the wine quality level aimed to be produced from the specific vineyard. While severe water deficit levels are related to improved wine quality, they may lead to a yield decrease [9,12,16,17,18,19,20]. Drought stress is considered to raise grape quality by two principal means. The first is berry size reduction, which leads to an increase in the skin to pulp ratio which in turn results in an increase in the color and aroma compounds in the must [8,9,12,21]. The second is by accelerating the metabolic pathways of color and aroma compounds [11,21,22,23].

Imposing the proper drought stress at the suitable phenological stage may raise wine quality with almost no yield reduction [9,14,24,25,26]. Tempranillo vines showed phenological sensitivity to drought stress in a way that affected berry quality (Girona et al., 2009). Post-veraison water application increased the must sugar level and wine alcohol content in Tempranillo vines. However, drought stress during the pre-veraison period led to higher concentrations of phenols and anthocyanins (Intrigliolo and Castel, 2010). On the other hand, inducing incorrect drought stress during a specific phenological stage may lead to a significant yield loss, and in some cases, even to decreases in sugar accumulation, and anthocyanin and polyphenol concentrations in berries [9,11,12,17,18,20,27]. In the long term, extended severe drought stress conditions may lead to a decrease in vegetative growth and shorten the vineyard’s sustainability. One of the methods for applying deficit irrigation is referred to as ‘regulated deficit irrigation’ (RDI). The RDI method is based on the implementation of dynamic water stress coefficients over the irrigation phase, leading to variance in the drought stress levels along the growing season [9,14,16,24,27,28,29]. The logic of the RDI method is that vines have diverse reactions to drought stress levels across the growing season, so, if wisely imposed, the method may lead to balanced vegetative growth and a decrease in berry size, while causing only minor yield loss [8,9,12,13,14,20,25,30].

In past experiments, we have shown that an irrigation regime in which a high water availability is maintained during stage I of berry development [31], followed by drought stress application at stages II and III (as compared to imposing consistent drought stress throughout all of the growing season) leads to enhanced vegetative growth, increased annual ring growth, and specific hydraulic conductivity, while preserving higher yields and an improved wine quality [9,13]. In order to achieve a better understanding of the effect of improved water availability during stage I of berry development on grapevine vegetative growth and yield parameters, we conducted a trial in which we examined a method for determining the appropriate irrigation initiation timing. This trial was a part of our effort to develop a skilled irrigation model for wine vineyard growing [9,13,20,32,33]. The irrigation of different treatments in the above experiment was initiated at decreasing levels of midday stem water potentials (−0.6, −0.8, −1.0, −1.2 MPa), corresponding to delayed dates of irrigation initiation and lower total water amounts applied, respectively. We showed that a high water availability during stage I (the vegetative growth period during spring) enhanced vegetative growth and increased yield levels, whereas withholding irrigation until the advanced periods of the growing season resulted in early drought stress, which in turn decreased vegetative growth, altered photosynthesis and gas exchange parameters, and lowered yield levels [9,20].

In the current work, we examined the effects of irrigation initiation timing on vine daily stem water potential and the consequences on the quality attributes of the produced wines. We show a detailed chemical and organoleptic analysis of the wines, and discuss the implications for quality wine vineyards growing in semi-arid areas.

## 2. Materials and Methods

### 2.1. Experimental Site and Design

This experiment was conducted in the commercial “Kida” vineyard (759 m asl), located in the central mountain district of Israel (lat. 32.2° N, long. 35° E), a premium wine-growing region. The climate that prevails in the vineyard is characterized as semi-arid, with most of the precipitation occurring during wintertime (84% winter rainfall, annual average of 415 mm), and fairly cool nights (minimum < 20 °C) combined with hot days (maximum > 30 °C). The soil at the experimental site is deep, stone-free terra rossa (36.4% sand, 30.6% silt, and 33% clay). The vineyard was planted during 2007 with *Vitis vinifera. Vitis vinifera* cv. ‘cabernet sauvignon’ vines were grafted onto 110 Richter rootstock and oriented north-west/south-east (115°). The vineyard density was 3 m between rows and 1.5 m between adjacent vines (2222 vines per hectare). The vines were trained onto a 2-m-high vertical trellis system (VSP) with two foliage wires, designed as bi-lateral cordons, and winter pruned to 16 spurs (each with two buds). The design of the experiment was a randomized complete block, with four replicates of the five irrigation treatments, as suggested by van Es et al. (2007) [34]. Each replicate/block consisted of one data row surrounded by additional two border rows. Each replicate plot comprised 16 vines (12 measurement vines and two border vines from each side). Three representative vines from each plot were marked and used for physiological and vegetative measurements. Except for irrigation, the vineyard was treated following the local agrotechnical procedures (hedging and pruning treatments, weed control, pest management). The meteorological data used for the irrigation calculations was obtained from a local automatic meteorological station (located inside the vineyard). Information about the meteorological station, vineyard design, and irrigation system and appliance has been published in the past [2,3].

### 2.2. Irrigation Treatments, Calculation of Irrigation Amounts

**Figure 1:**
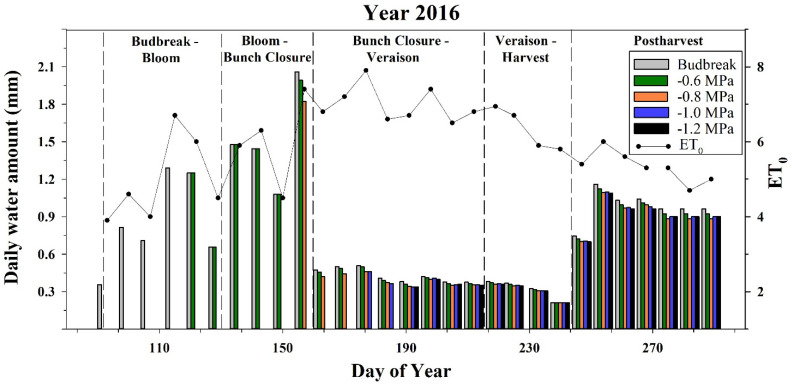
Daily irrigation water amounts (mm/day) applied once a week, and weekly average of reference evapotranspiration (ET_o_) calculated according to the Penman–Monteith equation. Kida vineyard, ‘cabernet sauvignon’ 2016.

Irrigation initiation timing was set individually per treatment according to its midday stem water potential (Ψ_s_) threshold (excluding budbreak treatment) (Figure 1). Irrigation was initiated at budbreak in the first treatment, whereas in the other four treatments it began when Ψ_s_ values fell under the threshold of: −0.6, −0.8, −1.0, or −1.2 MPa. In all irrigated treatments, water amounts were calculated as the percentage of crop evapotranspiration (ET_c_). The calculation of the ET_c_ was conducted following the equation: ET_c_ = ET_o_ × K_c_. The reference evapotranspiration (ET_o_) was calculated according to the Penman–Monteith equation [35] using meteorological data obtained from the adjacent meteorological station. The crop coefficient (K_c_) values were calculated according to the leaf area index–K_c_ relationship, as previously described [36]. The growing season was divided into three stages of berry development, following Kennedy et al. (2002): stage I from bloom until bunch closure, stage II from bunch closure up to veraison, and stage III from veraison until harvest. The water amounts were applied once a week to irrigated treatments at a rate of 40% of ET_c_ throughout stage I of berry development, 15% of ET_c_ at stage II of berry development, and 10% in the course of stage III of berry development.

### 2.3. Midday Stem Water Potential and Yield Measurements

Every week (before irrigation) at solar noon, Ψ_s_ was measured using a pressure chamber (model Arimad 3000, MRC, Hulon, Israel), in order to define each treatment irrigation initiating threshold. At the end of each of the three growing stages, daily measurements (06:00 to 19:00) of Ψ_s_ were conducted. Twelve fully expanded, sunlit, and mature leaves from each treatment (3 per replicate × 4 replicates) were kept in plastic bags covered with aluminum foil for 2 h prior to measurement [2].

When fruit’s must TSS level reached 24.5 °Brix, each plot was harvested separately. Within each plot, all of the 12 measurement vines were harvested (separately), and their number of clusters and total yields were recorded. The berry mass was determined by randomly picking and weighing 100 berries per treatment (25 per replicate × 4 replicates). The bunch mass was calculated by dividing the vine yield by the cluster number, and the number of berries per cluster was attained by dividing the bunch mass by the berry mass.

### 2.4. Wine Vinification

From each replicate, the total yield was taken for wine preparation (about 50 kg). Entire clusters were destemmed, crushed, and placed in 100 L stainless-steel always-full tanks. In order to initiate alcoholic fermentation, 10 g of the commercial *Saccharomyces cerevisiae* strain Clos (Lallemand Inc., Montreal, QC, Canada) was added. The cap punch down operations, for the extraction of color and polyphenols from the skins, were carried out three times a day during the 8 days of maceration in a temperature-controlled room at 25 °C. At day 9, the wine was separated from the pomace by pressing it using a hydraulic press, and kept at the same temperature until its density dropped below 0.994 g/mL. The dry young wine was decanted and left at 20 °C for malolactic fermentation. Following the completion of malolactic fermentation, sulfur dioxide was added to the wine (as potassium metabisulfite) at a concentration of 60 mg/L. The wine was decanted a week later and stored at 15 °C in 10 L demijohns. Two months later, 2 g/L of French oak wood chips (World Cooperage, Napa, CA, USA) was added to the demijohns, and left to age for an additional month. The chips’ addition aimed to give a touch of wood, which from our experience helps the tasters to assess the quality and aging ability of the wine. The wine was racked again, and bottled in 750 mL dark wine bottles until further analysis.

### 2.5. Must and Wine Analysis

Must samples were prepared from all harvested grapes by crushing. The juice was left to settle for an hour, and then decanted. The basic parameters of must are total soluble solids (TSS, °Brix), total acidity (TA), and pH. For TSS determination, a digital refractometer was used (Pocket PAL-1, ATAGO, Tokyo, Japan). TA and pH were measured by a Hanna HI 2211 pH meter (Hanna Instruments Inc, Woonsocket, RI, USA). TA was expressed as the concentration of tartaric acid (g/L), and was determined by diluting 10 mL of must with 10 mL of distilled water and subsequently titrating with 0.1 M NaOH to pH 8.2, according to the following calculation: TA = (Vol._NaOH_ × 0.75), where Vol._NaOH_ is the volume of added NaOH. The basic parameters of wine samples (concentration of ethanol and sugars, TA, pH, and concentrations of tartaric, malic, and acetic acids) were determined by the software “OenoFoss” (FOSS, Hilleroed, Denmark). A Super Dee digital distillator and Super Alcomat electronic hydrostatic balance (Gibertini, Milano, Italy) were used to validate the levels of alcohol and VA.

Basic wine analysis was conducted to determine the alcohol, TA, and pH levels. The wines of all treatments were within a narrow range of alcohol (13.4–13.7), with no differences between treatments. TA levels were also within a narrow range of 6.6–6.8, and pH levels were between 3.79 and 3.81. The volatile acidity and residual sugars were within the acceptable ranges for red wine, with no significant trend between treatments. Basic wine color analysis was conducted using spectrophotometric analysis, with absorbance measurements at 420 nm, 520 nm, and 620 nm. The color intensity was calculated by combining the AU scores of all three wavelengths, and the color hue by the ratio of 420:520 nm.

### 2.6. Wine Sensory Evaluation

Following wine stabilization and initial aging for 6–8 months, the wines were blind tasted by a qualified panel of eight professional oenologists, for their sensory quality attributes. The panel blindly tasted 20 young wines, four from each treatment (one per replicate). The four tasting flights were set such that every flight included one repeat of every treatment, in a randomized manner. The sensory evaluation was conducted using a method adopted from that of the OIV (international organization of vine and wine) score sheet for dry red wines [37] Briefly, the total wine sensory score sums up to 100 points, combined from color—”visual”—including color quality (up to 5 points) and color intensity (up to 10); smell—“nose”—including aroma intensity (up to 8), aroma genuineness (up to 6), and aroma quality (up to 16); “taste”—including taste intensity (up to 8), taste genuineness (up to 6), taste quality (up to 22), and after-taste (up to 8); and general harmony (up to 11).

### 2.7. Determination of Polyphenols

In general, the determination of total and specific polyphenols was conducted using the method developed in our laboratory, as published by Rosenzweig et al. (2017) [38]. Briefly, the total phenolic determination method was based on spectrophotometric analysis, with absorbance measurements at 280 nm, using a calibration curve of gallic acid to obtain the results in mg gallic acid/L. Specific phenolics were determined following sample preparation by filtration through a 0.22 μm PTFE membrane filter and separation by C18 reverse phase column by HPLC. Measurements were conducted: at 280 nm for the detection of gallic acid, catechin, epicatechin, vanillin, and p-coumaric acid; at 257 nm for the detection of vanillic acid and quercetin; and at 325 nm for the detection of caffeic acid. The identification and quantification of specific peaks was conducted using appropriate standards’ calibration curves.

### 2.8. Determination of Glycosylated Anthocyanins

The analysis protocol was based on de Andrade et al. [39]. Chemicals and reagents: deionized water was purified with a Milli-Q water system, acetonitrile of HPLC grade was obtained from BioLab (Ashkelon, Israel), formic acid of analytical reagent grade was obtained from Merck, and the anthocyanins cyanidin-3-*O*-glucoside, delphinidin-3-*O*-glucoside, peonidin-3-*O*-glucoside, petunidin-3-*O*-glucoside chloride, and malvidin-3-*O*-glucoside were supplied by Extrasynthese.

Standard solutions: stock solutions of anthocyanins were prepared by dissolving these compounds in 12% ethanol and 88% water, and adjusting to pH 3 with formic acid to a final concentration of 100 ppm. Dilutions in the concentration range of 0.36–93 ppm were analyzed for the calculation of calibration curves.

Sample preparation and analysis: twenty samples of red wines produced from the grapes of the different treatments were analyzed. A triplicate of each sample was run for analysis. The samples were filtered through a 0.22 μm PTFE membrane filter. The analyses were performed in duplicate immediately after wine bottle opening.

The HPLC analyses were carried out using a Jasco HPLC, composed of a UV/Vis detector (UV-4070), RHPLC pump (PU-4180), column oven (CO-4060), and RHPLC autosampler (AS-4150), from Extrema. The chromatographic separation of anthocyanins was performed on a reversed phase column Luna Omega 250 mm × 4.6 mm I.D. 5 μm 100 A (Phenomenex, Torrance, CA, USA).

Analytical Conditions: the mobile phase was a gradient of water/acetonitrile (87:3) (solvent A) and water/acetonitrile (40:50) (solvent B), both adjusted to pH 1.9 with formic acid, at a flow rate of 1.8 mL/min. Detection was performed at 520 nm. The temperature of the column oven was 40 °C. The following gradient was used: 0 min, 6% B; 0–15 min, 6–30% B; 15–16 min, 30–100% B; 16–20 min, 100% B; 20–21 min, 100–6% B; 21–26 min, 6% B. The peak areas and concentrations of compounds from the chromatograms were analyzed.

### 2.9. Statistical Analyses

One-way ANOVA was conducted, followed by the Tukey post hoc test, for analyzing the difference among means of yield, must, and wine characteristic in each replicated plot (4 plots per treatment) using JMP Pro 15.1.0 Statistical Software (SAS Institute Inc., Cary, NC, USA) to determine the statistical significance of differences between the treatment means at α = 0.05.

## 3. Results and Discussion

### 3.1. Irrigation Application

Each of the five treatments was irrigated differentially according to the research plan at budbreak (DOY 94), and when midday stem water potential (Ψs) reached values of −0.6, −0.8, −1.0, and −1.2 MPa, at two weeks prior to bloom (DOY 122), bunch closure (DOY 158), initiation of veraison (DOY 178), and end of veraison (DOY 194), respectively. The ET_o_ varied dramatically between these phenological stages and was approximately 4 mm/day at the bud break stage, 5 mm/day at the bunch closure stage, and 7 mm/day between the bunch closure and veraison, dropping again to an average of 5 mm/day between the veraison and harvest (Figure 1).

The water stress coefficients (40%, 15%, 10%) used in the current research over the different phenological stages represent the optimal combination for quality vineyard RDI strategy, as found by our group in past works [9,13]

The annual water amounts applied were 113 mm/season for the budbreak treatment (Table 1), and 20 mm/season for the late irrigation treatment (−1.2 MPa). These results represent the ranges of common practice for premium vineyard irrigation in Israel, and are in accordance with the reported deficit irrigated vineyards found in similar climate regions [23,25,27,40,41,42,43,44]

The gradual decrease in the amount of water applied during the different treatments at each irrigation timepoint was due to the fact that, in the current study, the irrigation method was based on the leaf area index-crop coefficient relationship. Treatments irrigated earlier maintained higher leaf area index (LAI) levels, as shown by Munitz et al. [20]. The phenomenon of improved LAI levels as a result of improved water availability during stage I and II, and the effects of LAI levels on the vine’s water consumption are well established [9,13,20,33,45,46]. Lately, the relative influences of LAI and meteorological variables on cabernet sauvignon water consumption were found to be 69.4% and 30.6%, respectively [45,47].

### 3.2. Vine Water Status

**Figure 2:**
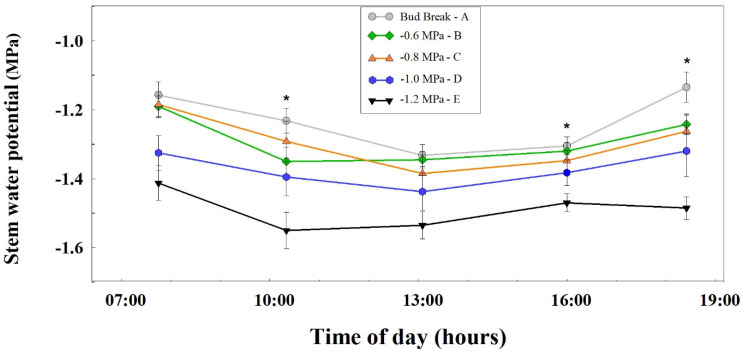
The daily pattern of midday stem water potential (SWP) of vines exposed to different irrigation treatments during the end of stage II at 2016 (01/08/16). Each value is the mean of 12 leaves (four replicates and three leaves per replicate). The bars denote one standard error of the mean. Asterisks indicate significant differences (*p < 0.05*) between irrigation treatments according to Tukey’s test. Measurements were taken on the day before irrigation was applied, Kida vineyard, ‘cabernet sauvignon’ 2016.

The midday stem water potential (SWP) is a very sensitive indicator of vine water status [9,20,40,48,49,50], hence it was chosen in the current study to serve as a threshold for irrigation initiation. The full 2016 seasonal SWP pattern has been previously published [2]. At the end of stage II (01/08/16), following the irrigation initiation of all treatments, the recording of the daily pattern of midday SWP of the vines exposed to the different irrigation regimes was conducted. This analysis revealed that, although all the treatments were irrigated with similar amounts of water at that time, the SWP values measured for the −1.2 MPa treatment were the lowest (~−1.4 MPa) at day-break, dropping to −1.55 MPa at noon, and recovering to −1.5 MPa in the afternoon. The treatment of −1.0 MPa showed a slightly improved water potential, starting around −1.3 MPa, dropping to −1.4 MPa, and recovering to −1.3 MPa in the afternoon. The early irrigated treatments (bud break, −0.6, −0.8 MPa) showed improved (relative to previous) and similar daily trends, starting the day at −1.2 MPa, dropping to about −1.35 MPa, and recovering to −1.2 MPa in the afternoon. (Figure 2). A SWP value of −1.4 MPa is considered to be the threshold level of severe drought stress in vineyards, which is required for the production of high quality red wine grapes [20,31,51]. Our results show that only the −1.2 and −1.0 MPa irrigation initiation treatments exhibited lower values of SWP than this threshold. We propose that the more negative SWP of the later irrigated plots at the time of measurement was due to the generally lower amount of water available for the vines (due to the lower cumulative amount of water applied at irrigation, and lower water availability in the soil).

As stated previously, drought stress can affect berry composition in two ways: directly, by enhancing the expression of genes along the metabolic pathways of aroma and color compounds, and indirectly, by reducing berry size [32,52,53,54,55]. In shiraz berries, drought stress was found to directly regulate the last steps of the anthocyanin metabolic pathway [22]. Another direct effect of drought stress on anthocyanins synthesis was observed in merlot vines [53,54]. The direct effect of drought stress on polyphenol synthesis was recorded in shiraz and cabernet sauvignon vines [55,56]. Polyphenols are involved in plant adaptations to stress, and are known to be part of the defense mechanism against reactive oxygen species, thus, their production is usually increased as a response to environmental stresses (such as drought) affecting photosynthetic reactions [57]. As a consequence of the above, there is an evolutionary logic for plants to enhance the concentration of polyphenols with antioxidant activity, as a response to drought stress. We have shown that in the current research, drought stress indirectly affected berry composition by considerably reducing berry weight [20]. Nevertheless, the direct effect of drought stress on berry composition is also possible in our case, and, it is most likely that both mechanisms are involved in berry composition variation, as found by others [53,55].

### 3.3. Yield Parmeters

Following harvesting of the grapes, the measurements of yield per vine presented a significant gradual decrease in accordance to the level of SWP at the time of irrigation initiation, with 7.77 kg/vine for the budbreak treatment, and down to 5.37 kg/vine for the −1.2 MPa treatment (Table 2). Similar significant differences were recorded for the bunch mass and 100 berry mass. The number of bunches per vine, and berries per bunch showed similar trends, but these results were not found to be statistically significant (Table 2). These findings are in accordance with results shown in previous work, demonstrating the positive effect of improved SWP during stage I on number of clusters, yield, and berry and cluster weights [9,13,20,33,45,46].

### 3.4. Must Parmeters

Harvest was conducted for each replicate when the average sugar level reached approximately 24.5 °Brix. All treatments were harvested on the same day (30 August 2016), with no significant differences in major must components (Table 3).

### 3.5. Wine Parmeters

Following the grapes’ harvest and processing into wine by micro vinification, an analysis of wine quality parameters and chemical composition was conducted. We conducted the deep analysis on the finished wine, rather than on grapes, in order to assess the actual impact of the irrigation treatments on a final wine’s characteristics, which is more relevant for application to the wine industry. The analysis of wine color conducted by spectrometry revealed a significant gradual increase in the yellow, red, and blue coloration levels of the wines, in accordance with the decrease in the level of SWP at the irrigation initiation time. Thus, the levels of the wine produced from the budbreak treatment were found to be 3.5, 4.4, and 1.2 for the yellow, red, and blue colorations, respectively, and 4.2, 5.7, and 1.6 for the −1.2 MPa treatment (Table 4). Consequently, the color intensity (CI) levels followed the same significant gradual increase trend.

Interestingly, the color hue (CH) levels followed a reversed pattern, with the higher levels found for the budbreak treatment and lower levels for the late-irrigated treatments. This pattern results from a higher increase in red and blue coloration levels than those of the yellow coloration for the postponed irrigation treatments. Finally, the total phenolic material levels were significantly higher for the −1.2 MPa treatment compared to the budbreak treatment (Table 4). These results indicate that delayed irrigation, causing a deeper drought stress at early stages [20] and an accumulative intrinsic water shortage at later stages of ripening, has a positive effect on both the total coloration and phenolic levels. Other works applying RDI by many different strategies have shown similar trends of higher coloration and chroma in the stressed treatments. For example, one work assessing the outcome of RDI, by the application of 60%, 70%, 80%, and 100% (traditional drip irrigation) of the estimated evapotranspiration (ET_c_), found that vines irrigated by 60% had increased anthocyanin levels in both grapes and wine [58,59]. This same work, on the other hand, found a decrease in TA and increase in pH in the 60% RDI treatment, in contrast to our findings. Another work on cabernet sauvignon, applying milder drought stress conditions (up to −1 MPa) also showed an increase in total anthocyanins and chroma for the stress versus unstressed treatments [60]. The causes of these effects might be direct—by a shift in the molecular pathways in the grape (caused by relatively negative stem water potential), leading to the facilitated synthesis of phenolic compounds including anthocyanins and procyanidins [8,9,12,21]. Another option is an indirect manner—due to the decrease in berry size, which results in a higher skin/pulp ratio [11,20,23,57].

An analysis of the main forms of anthocyanins (Table 5) and main phenolic compounds (Table 6) was conducted using HPLC. The levels of malvidin-3-*O*-glucoside, the main anthocyanin in wine grapes, showed a tendency to rise with the decrease in SWP at irrigation initiation. A significant increase in the malvidin-3-*O*-glucoside level (1.4 fold) was found between the treatment irrigated at budbreak and that of −1.2 MPa. Delphinidin-3-*O*-glucoside, petunidin-3-*O*-glucoside, and peonidin-3-*O*-glucoside levels showed an even more dramatic response, with additional treatments showing significant higher levels compared to the bud break treatment, and increased 2.4, 1.9, and 1.4 fold between the −0.6 MPa and −1.2 MPa treatments, respectively. The levels of cyanidin-3-*O*-glucoside showed no clear response to the irrigation treatment. Similar results showing higher amounts of most primary anthocyanins following RDI treatments were shown in other works on cabernet sauvignon [61,62,63]. One of these works [60] also showed that cyanidin-3-*O*-glucoside is indifferent to RDI treatments, and the dose did not accumulate following water deficits of higher levels.

Wine includes an array of phenolic compounds, the majority of which originate in the grape. They play a significant role in wine, influencing astringency and bitterness particularly in red wines. Additionally, phenolics are natural antioxidant compounds [64], and are important for red wine’s color stability. Phenolics function as a natural wine preservative, and are the foundation for extended aging [65].

Our analysis of the monomeric phenolic compound levels in the wines (Table 6) revealed an apparent increase in the levels of catechin, epicatechin, vanillin, quercetin, and vanillic acid, when irrigation was initiated later during the growing season. On the other hand, the level of p-coumaric acid was not affected by the irrigation treatment. Interestingly the caffeic acid levels showed a reverse trend, decreasing with the reduction in SWP values at the irrigation initiation day, from 8.2 in the budbreak treatment to 6.6 in the −1.2 MPa treatment. Nevertheless, the caffeic acid results were not found to be statistically significant (*p* > 0.05), due to high variation between replicates. No clear trend was found for the levels of gallic acid in the wines from various irrigation treatments. The alteration of specific phenolic compound’s concentrations in wine may be of nutraceutical importance. Catechin and epicatechin play a pivotal role in the maintenance of the body weight [66], and serve as neuroprotective [65] and cardioprotective agents [62]. Vanillin and vanillic acid provide a specific flavor and fragrance to wine, and have neuroprotective, anticarcinogenic, and antioxidant properties [63]. Meanwhile, quercetin is utilized as an antioxidant and nutraceutical compound [67], and is well known for its anti-inflammatory [68], cardioprotective [69], immunoprotective [68], anticancer [70,71], and antidiabetic [72] properties. Moreover, it may be also utilized as a potential therapeutic agent against COVID-19 [73]. Multiple scientific works, including clinical trials and epidemiological studies show that moderate wine consumption, especially when combined with a ‘Mediterranean diet’ has multiple positive health effects, mainly attributed to the antioxidant properties of resveratrol and other wine polyphenolics (for a review, see [74]). Hence, we can conclude that an increase in the specific phenolics observed due to the delaying of irrigation initiation may serve to enhance the nutraceutical potential of the wines produced.

### 3.6. Wine Sensory Evaluation

Finally, a wine sensory evaluation was conducted by a panel of eight professional winemakers, using an OIV-based tasting method. We used this method, and not descriptive analysis or other scoring methods, in order to present the relevant industry standard scorings for the wines (Table 7). The color quality and intensity were significantly higher in the wines originating from the lower SWP levels at irrigation initiation day. Other attributes did not show a clear significant tendency, but, when summing the sensory evaluation score, a clear and significant increase in total wine score was found for the wines produced from grapes irrigated later in the season (−0.8, −1.0, −1.2 MPa), as compared to those irrigated earlier (budbreak, −0.6 MPa). These results are in agreement with those reported by Cáceres–Mella et al. (2018), which described wines produced from RDI-treated grapes as more intense in color, more fruity, and expressing fullness in the mouth [60]. The difference we show between the earliest irrigated treatment (budbreak) and that of the latest (−1.2 MPa) is of 4.5 points. This is a substantial difference in this scale, between a medium range wine (84.4), winning a silver medal, to a high grade wine (89.2), winning a high gold medal [37].

## 4. Conclusions

The results presented here show that postponing irrigation results in a lower intrinsic midday water potential. This is followed by lower yields, a lower number of bunches, and smaller berries. The wine produced following later irrigation has a higher color, higher total phenolics levels and a better color hue. The higher levels of color are caused by higher levels of most monomeric anthocyanins, but not of cyanidin-3-*O*-glucoside. Most phenolic compounds accumulated in higher levels in the wines of the postponed irrigation treatments, except those of gallic acid and p-coumaric acid, which had no clear trend, and that of caffeic acid, which showed an opposite trend. The final wines’ scores, measured by an industry relevant sensory evaluation method (OIV) were significantly higher for the late-irrigated treatments. Our practical conclusion from these results is that for premium winemaking, by delaying irrigation initiation in a skilled manner, a winery can lower yields and gain a higher quality wine, richer in color and taste compounds. In a wider aspect, this work and others dealing with irrigation regimes, mostly relevant today to classical warm and dry regions, may become relevant to wider areas in the near future, as climate change may result in increased drought stress occurrences. Further analysis should be carried out on wines during aging to assess the long-term implications of irrigation initiation treatments.

## Figures and Tables

**Table 1:** Dates and day of the year (in parentheses) of irrigation initiation point for each irrigation treatment, and total seasonal water amounts (mm) applied, Kida vineyard, ‘cabernet sauvignon’ 2016.

Treatment	Dates of Irrigation Initiation	Seasonal Irrigation (mm)
Budbreak	4 April (94)	113.1
−0.6 MPa	2 May (122)	92.5
−0.8 MPa	7 June (158)	44.6
−1.0 MPa	27 June (178)	26.5
−1.2 MPa	13 July (194)	20.1

**Table 2:** Yield components of each irrigation treatment. Kida vineyard, ‘cabernet sauvignon’ 2016. Different capital letters indicate significantly different treatments, *p* < 0.05.

Treatment	Yield (kg/Vine)	Bunch (Number/Vine)	Bunch Mass (gr)	100 Berry Mass (gr)	Berry (Number/Bunch)
Budbreak	7.77 A	59.0	133.5 A	138.5 A	83.5
−0.6 MPa	7.00 AB	56.5	125.2 B	136.6 A	86.0
−0.8 MPa	6.12 AB	55.7	109.7 AB	126.6 AB	77.1
−1.0 MPa	5.75 B	50.5	113 AB	120.7 B	77.2
−1.2 MPa	5.37 B	52.7	102.2 B	117.1 B	77.1

**Table 3:** Must composition parameters at harvest. Kida vineyard, ‘cabernet sauvignon’ 2016. Different letters indicate significantly different treatments, *p* < 0.05.

Treatment	TSS (°Brix)	pH	TA (g/L)
Budbreak	24.7 A	3.48 A	4.9 A
−0.6 MPa	24.2 A	3.48 A	4.3 A
−0.8 MPa	25.2 A	3.51 A	4.7 A
−1.0 MPa	24.9 A	3.46 A	5.0 A
−1.2 MPa	24.6 A	3.51 A	4.5 A

**Table 4:** Wine color and polyphenols general parameters. Yellow, red, and blue colorations were measured by spectrometer at 420, 520, and 620 nm respectively. CI (color intensity) is the sum of all 3 wavelengths. CH (color hue) is the ratio of 420:520 nm absorptions. Total phenolics were measured by spectrometer at 280 nm. Different letters indicate significantly different treatments, *p* < 0.05.

Treatment	Yellow, AU	Red, AU	Blue, AU	CI, AU	CH	Total Phenolics, (as mg Gallic Acid/L)
Budbreak	3.47 B	4.38 C	1.25 B	9.12 B	0.793 A	872 B
−0.6 MPa	3.61 AB	4.70 CB	1.32 AB	9.63 AB	0.768 AB	925 AB
−0.8 MPa	3.68 AB	4.96 CBA	1.39 AB	10.03 AB	0.740 B	950 AB
−1.0 MPa	4.00 AB	5.45 BA	1.53 AB	10.98 AB	0.735 B	975 AB
−1.2 MPa	4.18 A	5.71 A	1.61 A	11.48 A	0.735 B	1026 A

**Table 5:** Wine anthocyanin contents (ppm), as measured by HPLC. The analysis was conducted on young wines fermented by micro vinification from 50 kg ‘cabernet sauvignon’ grapes following the irrigation trial in 2016. Different letters indicate significantly different treatments, *p* < 0.05.

Treatment	Malvidin-3-*O*-glucoside	Delphinidin-3-*O*-glucoside	Cyanidin-3-*O*-glucoside	Petunidin-3-*O*-glucoside	Peonidin-3-*O*-glucoside
Budbreak	66.1 B	0.36 B	0.69	4.91 B	2.12 C
−0.6 MPa	78.7 AB	0.34 B	0.77	4.91 B	2.35 CB
−0.8 MPa	90.2 AB	0.50 AB	0.65	7.60 AB	2.87 AB
−1.0 MPa	84.5 AB	0.59 AB	0.71	7.68 A	2.81 AB
−1.2 MPa	94.2 A	0.83 A	0.79	9.08 A	3.21 A

**Table 6:** Wine polyphenolic contents (ppm), as measured by HPLC. The analysis was conducted on young wines fermented by micro vinification from 50 kg ‘cabernet sauvignon’ grapes following the Irrigation trial in 2016. Different letters indicate significantly different treatments, *p* < 0.05.

Treatment	Gallic Acid	Catechin	Epicatechin	Vanillin	p-Coumaric Acid	Vanillic Acid	Quercetin	Caffeic Acid
Budbreak	20.02	116.5 B	48.1 B	2.24 B	4.33	2.27 B	1.15 B	8.23
−0.6 MPa	17.69	142.3 AB	53.3 AB	2.68 AB	4.24	2.36 AB	1.31 B	7.44
−0.8 MPa	21.53	154.5 AB	48.7 B	3.76 A	4.26	2.83 AB	1.69 AB	7.46
−1.0 MPa	24.54	144.3 AB	49.6 B	3.24 AB	4.37	2.94 A	1.62 AB	6.91
−1.2 MPa	26.96	162.8 A	56.2 A	3.55 A	4.47	2.99 A	2.04 A	6.60

**Table 7:** Wine sensory evaluation results. The average scores for color, smell, taste, and harmony (general) parameters, as well as the total scores given to the wines by the tasting panel, are presented. The sensory evaluation was based on the OIV method. Different letters indicate significantly different treatments, *p* < 0.05.

Treatment	Color Quality	Color Intensity	Smell Intensity	Smell Genu	Smell Quality	Taste Intensity	Taste Genu	Taste Quality	After Taste	General	Total Score
Budbreak	3.9 BC	7.8 B	6.8	4.9	13.8	6.8	4.9 AB	18.4	6.9	9.9	84.4 C
−0.6 MPa	3.6 C	8.2 B	7.0	4.8	13.8	6.8	4.6 B	19.0	6.8	9.9	84.7 BC
−0.8 MPa	4.2 B	8.5 B	6.9	5.1	14.1	7.0	5.0 AB	18.9	7.0	9.9	86.8 AB
−1.0 MPa	4.3 B	8.5 B	6.9	4.6	14.0	6.9	4.8 AB	19.3	6.7	9.8	86.0 AB
−1.2 MPa	4.8 A	9.6 A	7.2	5.0	14.0	7.2	5.1 A	19.1	7.0	10.0	89.2 A

## Data Availability

Not applicable.

## References

[1] Drappier J., Thibon C., Rabot A., Geny-Denis L. (2019). Relationship between wine composition and temperature: Impact on Bordeaux wine typicity in the context of global warming—Review. Crit. Rev. Food Sci. Nutr..

[2] Collins M.J., Knutti R., Arblaster J., Dufresne J.-L., Fichefet T., Friedlingstein P., Gao X., Gutowski W.J., Johns T., Krinner G., Stocker T.F., Qin D., Plattner K., Tignor M., Allen S.K., Boschung J., Nauels A., Xia Y., Bex V., Midgley P.M. (2013). Long-term climate change: Projections, commitments and irreversibility. Climate Change 2013 the Physical Science Basis: Working Group I Contribution to the Fifth Assessment Report of the Intergovernmental Panel on Climate Change.

[3] Hannah L., Roehrdanz P.R., Ikegami M., Shepard A.V., Shaw M.R., Tabor G., Zhi L., Marquet P.A., Hijmans R.J. (2013). Climate change, wine, and conservation. Proc. Natl. Acad. Sci. USA.

[4] Kizildeniz T., Mekni I., Santesteban H., Pascual I., Morales F., Irigoyen J.J. (2015). Effects of climate change including elevated CO2 concentration, temperature and water deficit on growth, water status, and yield quality of grapevine (*Vitis vinifera* L.) cultivars. Agric. Water Manag..

[5] Parry M.L., Canziani O., Palutikof J., Van der Linden P., Hanson C. (2007). Climate Change 2007: Impacts, Adaptation and Vulnerability. Contribution of Working Group II to the Fourth Assessment Report of the Intergovernmental Panel on Climate Change.

[6] Matthews M.A., Ishii R., Anderson M.M., O’Mahony M.O. (1990). Dependence of wine sensory attributes on vine water status. J. Sci. Food Agric..

[7] Roby G., Harbertson J.F., Adams D.A., Matthews M.A. (2004). Berry size and vine water deficits as factors in winegrape composition: Anthocyanins and tannins. Aust. J. Grape Wine Res..

[8] Bravdo B., Hepner Y., Loinger C., Cohen S., Tabacman H. (1985). Effect of irrigation and crop level on growth, yield and wine quality of Cabernet Sauvignon. Am. J. Enol. Vitic..

[9] Munitz S., Netzer Y., Schwartz A. (2016). Sustained and regulated deficit irrigation of field-grown Merlot grapevines. Aust. J. Grape Wine Res..

[10] Chaves M.M., Santos T.P., Souza C.R., Ortuño M.F., Rodrigues M.L., Lopes C.M., Maroco J.P., Pereira J.S. (2007). Deficit irrigation in grapevine improves water-use efficiency while controlling vigour and production quality. Ann. Appl. Biol..

[11] Chaves M.M., Zarrouk O., Francisco R., Costa J.M., Santos T., Regalado A.P., Rodrigues M.L., Lopes C.M. (2010). Grapevine under deficit irrigation: Hints from physiological and molecular data. Ann. Bot..

[12] Keller M., Smithyman R.P.R.R.P., Mills L.J.L.J. (2008). Interactive effects of deficit irrigation and crop load on Cabernet Sauvignon in an arid climate. Am. J. Enol. Vitic..

[13] Netzer Y., Munitz S., Shtein I., Schwartz A. (2019). Structural memory in grapevines: Early season water availability affects late season drought stress severity. Eur. J. Agron..

[14] Girona J., Marsal J., Mata M., Del Campo J., Basile B. (2009). Phenological sensitivity of berry growth and composition of Tempranillo grapevines (*Vitis vinifera* L.) to water stress. Aust. J. Grape Wine Res..

[15] Smithyman R., Wample R. (2001). Water deficit and crop level influences on photosynthetic strain and blackleaf symptom development in Concord grapevines. Am. J. Enol. Vitic..

[16] Romero P., Fernández-Fernández J.I., Martinez-Cutillas A. (2010). Physiological thresholds for efficient regulated deficit-irrigation management in winegrapes grown under semiarid conditions. Am. J. Enol. Vitic..

[17] Grimes D.W., Williams L.E. (1990). Irrigation Effects on Plant Water Relations and Productivity of Thompson Seedless Grapevines. Crop Sci..

[18] Medrano H., Escalona J.M., Cifre J., Bota J., Flexas J. (2003). A ten-year study on the physiology of two Spanish grapevine cultivars under field conditions: Effects of water availability from leaf photosynthesis to grape yield and quality. Funct. Plant Biol..

[19] Cifre J., Bota J., Escalona J.M., Medrano H., Flexas J. (2005). Physiological tools for irrigation scheduling in grapevine (*Vitis vinifera* L.). Agric. Ecosyst. Environ..

[20] Munitz S., Schwartz A., Netzer Y. (2020). Effect of timing of irrigation initiation on vegetative growth, physiology and yield parameters in Cabernet Sauvignon grapevines. Aust. J. Grape Wine Res..

[21] Castellarin S.D., Matthews M.A., Di Gaspero G., Gambetta G.A. (2007). Water deficits accelerate ripening and induce changes in gene expression regulating flavonoid biosynthesis in grape berries. Planta.

[22] Ollé D., Guiraud J.L., Souquet J.M., Terrier N., Ageorges A., Cheynier V., Verries C. (2011). Effect of pre- and post-veraison water deficit on proanthocyanidin and anthocyanin accumulation during Shiraz berry development. Aust. J. Grape Wine Res..

[23] Zarrouk O., Francisco R., Pinto-Marijuan M., Brossa R., Santos R.R., Pinheiro C., Costa J.M., Lopes C., Chaves M.M. (2012). Impact of irrigation regime on berry development and flavonoids composition in Aragonez (Syn. Tempranillo) grapevine. Agric. Water Manag..

[24] Intrigliolo D.S., Castel J.R. (2010). Response of grapevine cv. ‘Tempranillo’ to timing and amount of irrigation: Water relations, vine growth, yield and berry and wine composition. Irrig. Sci..

[25] Romero P., Gil-Muñoz R., del Amor F.M., Valdés E., Fernández J.I., Martinez-Cutillas A. (2013). Regulated Deficit Irrigation based upon optimum water status improves phenolic composition in Monastrell grapes and wines. Agric. Water Manag..

[26] Williams L.E., Araujo F.J. (2002). Correlations among predawn leaf, midday leaf, and midday stem water potential and their correlations with other measures of soil and plant water status in *Vitis vinifera*. J. Am. Soc..

[27] Santesteban L.G., Miranda C., Royo J.B. (2011). Regulated deficit irrigation effects on growth, yield, grape quality and individual anthocyanin composition in *Vitis vinifera* L. cv. “Tempranillo”. Agric. Water Manag..

[28] Fernandes-Silva A., Oliveira M., Paço T.A., Ferreira I., Ondrašek G. (2019). Deficit Irrigation in Mediterranean Fruit Trees and Grapevines: Water Stress Indicators and Crop Responses. Irrigation in Agroecosystems.

[29] Barbagallo M.G., Vesco G., Di Lorenzo R., Lo Bianco R., Pisciotta A. (2021). Soil and regulated deficit irrigation affect growth, yield and quality of ‘nero d’avola’ grapes in a semi-arid environment. Plants.

[30] Hardie W., Considine J. (1976). Response of grapes to water-deficit stress in particular stages of development. Am. J. Enol. Vitic..

[31] Kennedy J. (2002). Understanding grape berry development. Pract. Winer. Vineyard.

[32] Munitz S., Netzer Y., Shetin I., Schwartz A. (2018). Water availability dynamics have long-term effects on mature stem structure in *Vitis vinifera*. Am. J. Bot..

[33] Munitz S., Schwartz A., Netzer Y. (2019). Water consumption, crop coefficient and leaf area relations of a *Vitis vinifera* cv. “Cabernet Sauvignon” vineyard. Agric. Water Manag..

[34] Van Es H.M., Gomes C.P., Sellmann M., van Es C.L. (2007). Spatially-Balanced Complete Block designs for field experiments. Geoderma.

[35] Allen R.G., Pereira L.S., Raes D., Smith M. (1998). Crop Evapotranspiration-Guidelines for Computing Crop Water Requirements-FAO Irrigation and Drainage Paper 56.

[36] Netzer Y., Yao C., Shenker M., Bravdo B.A., Schwartz A. (2009). Water use and the development of seasonal crop coefficients for Superior Seedless grapevines trained to an open-gable trellis system. Irrig. Sci..

[37] International Organisation of Vine and Wine OIV Standard for International Wine Competitions and Spiritous Beverages of Vitivinicultural Origin (OIV-Concours 332A-2009). https://www.oiv.int/public/medias/1848/oiv-concours-332a-2009-fr-avec-signature.pdf.

[38] Rosenzweig T., Skalka N., Rozenberg K., Elyasiyan U., Pinkus A., Green B., Stanevsky M., Drori E. (2017). Red wine and wine pomace reduced the development of insulin resistance and liver steatosis in HFD-fed mice. J. Funct. Foods.

[39] De Andrade R.H.S., Do Nascimento L.S., Pereira G.E., Hallwass F., Paim A.P.S. (2013). Anthocyanic composition of Brazilian red wines and use of HPLC-UV-Vis associated to chemometrics to distinguish wines from different regions. Microchem. J..

[40] Acevedo-Opazo C., Ortega-Farias S., Fuentes S. (2010). Effects of grapevine (*Vitis vinifera* L.) water status on water consumption, vegetative growth and grape quality: An irrigation scheduling application to achieve regulated deficit irrigation. Agric. Water Manag..

[41] Buesa I., Pérez D., Castel J., Intrigliolo D., Castel J. (2017). Effect of deficit irrigation on vine performance and grape composition of *Vitis vinifera* L. cv. Muscat of Alexandria. Aust. J. Grape Wine Res..

[42] Shellie K. (2017). Above Ground Drip Application Practices Alter Water Productivity of Malbec Grapevines under Sustained Deficit. J. Agric. Sci..

[43] Chacón-Vozmediano J.L., Martínez-Gascueña J., García-Navarro F.J., Jiménez-Ballesta R. (2020). Effects of water stress on vegetative growth and ‘merlot’ grapevine yield in a semi-arid mediterranean climate. Horticulturae.

[44] Buesa I., Intrigliolo D.S., Castel J.R., Vilanova M. (2021). Influence of water regime on grape aromatic composition of Muscat of Alexandria in a semiarid climate. Sci. Hortic. (Amst.).

[45] Ohana-Levi N., Munitz S., Ben-Gal A., Schwartz A., Peeters A., Netzer Y., Schwarz A., Peeters A., Netzer Y., Schwartz A. (2020). Multiseasonal grapevine water consumption—Drivers and forecasting. Agric. For. Meteorol..

[46] Williams L.E., Ayars J.E. (2005). Grapevine water use and the crop coefficient are linear functions of the shaded area measured beneath the canopy. Agric. For. Meteorol..

[47] Ohana-Levi N., Munitz S., Ben-Gal A., Netzer Y. (2020). Evaluation of within-season grapevine evapotranspiration patterns and drivers using generalized additive models. Agric. Water Manag..

[48] Choné X., Van Leeuwen C., Dubourdieu D., Gaudillere J.P. (2001). Stem water potential is a sensitive indicator of grapevine water status. Ann. Bot..

[49] Patakas A., Noitsakis B., Chouzouri A. (2005). Optimization of irrigation water use in grapevines using the relationship between transpiration and plant water status. Agric. Ecosyst. Environ..

[50] Santesteban L.G., Miranda C., Marín D., Sesma B., Intrigliolo D.S., Mirás-Avalos J.M., Escalona J.M., Montoro A., de Herralde F., Baeza P. (2019). Discrimination ability of leaf and stem water potential at different times of the day through a meta-analysis in grapevine (*Vitis vinifera* L.). Agric. Water Manag..

[51] Mirás-avalos J.M., Intrigliolo D.S. (2017). Grape Composition under Abiotic Constrains: Water Stress and Salinity. Front. Plant Sci..

[52] Castellarin S., Pfeiffer A., Sivilotti P., Degan M., Peterlunger E., Di Gaspero G. (2007). Transcriptional regulation of anthocyanin biosynthesis in ripening fruits of grapevine under seasonal water deficit. Plant Cell Environ..

[53] Savoi S., Herrera J.C., Carlin S., Lotti C., Bucchetti B., Peterlunger E., Castellarin S.D., Mattivi F. (2020). From grape berries to wines: Drought impacts on key secondary metabolites. Oeno One.

[54] Cook M.G., Zhang Y., Nelson C.J., Gambetta G., Kennedy J.A., Kurtural S.K. (2015). Anthocyanin composition of merlot is ameliorated by light microclimate and irrigation in central California. Am. J. Enol. Vitic..

[55] Hochberg U., Degu A., Cramer G.R., Rachmilevitch S., Fait A. (2015). Cultivar specific metabolic changes in grapevines berry skins in relation to deficit irrigation and hydraulic behavior. Plant Physiol. Biochem..

[56] Deluc L.G., Grimplet J., Wheatley M.D., Tillett R.L., Quilici D.R., Osborne C., Schooley D.A., Schlauch K.A., Cushman J.C., Cramer G.R. (2007). Transcriptomic and metabolite analyses of Cabernet Sauvignon grape berry development. BMC Genom..

[57] Stagnari F., Galieni A., Pisante M. (2016). Drought stress effects on crop quality. Water Stress Crop Plants A Sustain. Approach.

[58] Ju Y., Yang B., He S., Tu T., Min Z., Fang Y., Sun X. (2019). Anthocyanin accumulation and biosynthesis are modulated by regulated deficit irrigation in Cabernet Sauvignon (*Vitis vinifera* L.) grapes and wines. Plant Physiol. Biochem..

[59] Duan B., Ren Y., Zhao Y., Merkeryan H., Su-Zhou C., Li Y., Mei Y., Liu X. (2021). An adequate regulated deficit irrigation strategy improves wine astringency perception by altering proanthocyanidin composition in Cabernet Sauvignon grapes. Sci. Hortic. (Amst.).

[60] Cáceres-Mella A., Ribalta-Pizarro C., Villalobos-González L., Cuneo I.F., Pastenes C. (2018). Controlled water deficit modifies the phenolic composition and sensory properties in Cabernet Sauvignon wines. Sci. Hortic. (Amst.).

[61] Mandel S., Youdim M.B.H. (2004). Catechin polyphenols: Neurodegeneration and neuroprotection in neurodegenerative diseases. Free Radic. Biol. Med..

[62] Bertelli A.A.A., Das D.K. (2009). Grapes, wines, resveratrol, and heart health. J. Cardiovasc. Pharmacol..

[63] Arya S.S., Rookes J.E., Cahill D.M., Lenka S.K. (2021). Vanillin: A review on the therapeutic prospects of a popular flavouring molecule. Adv. Tradit. Med..

[64] Brewer M.S. (2011). Natural antioxidants: Sources, compounds, mechanisms of action, and potential applications. Compr. Rev. Food Sci. Food Saf..

[65] Waterhouse A.L. (2002). Wine phenolics. Ann. N. Y. Acad. Sci..

[66] Hursel R., Westerterp-Plantenga M.S. (2013). Catechin- and caffeine-rich teas for control of body weight in humans. Am. J. Clin. Nutr..

[67] Boots A.W., Haenen G.R.M.M., Bast A. (2008). Health effects of quercetin: From antioxidant to nutraceutical. Eur. J. Pharmacol..

[68] Li Y., Yao J., Han C., Yang J., Chaudhry M.T., Wang S., Liu H., Yin Y. (2016). Quercetin, inflammation and immunity. Nutrients.

[69] Patel R.V., Mistry B.M., Shinde S.K., Syed R., Singh V., Shin H.S. (2018). Therapeutic potential of quercetin as a cardiovascular agent. Eur. J. Med. Chem..

[70] Murakami A., Ashida H., Terao J. (2008). Multitargeted cancer prevention by quercetin. Cancer Lett..

[71] Rauf A., Imran M., Khan I.A., ur-Rehman M., Gilani S.A., Mehmood Z., Mubarak M.S. (2018). Anticancer potential of quercetin: A comprehensive review. Phyther. Res..

[72] Aguirre L., Arias N., Macarulla M.T., Gracia A., Portillo M.P. (2011). Beneficial effects of quercetin on obesity and diabetes. Open Nutraceuticals J..

[73] Agrawal P.K., Agrawal C., Blunden G. (2020). Quercetin: Antiviral significance and possible COVID-19 integrative considerations. Nat. Prod. Commun..

[74] Snopek L., Mlcek J., Sochorova L., Baron M., Hlavacova I., Jurikova T., Kizek R., Sedlackova E., Sochor J. (2018). Contribution of Red Wine Consumption to Human Health Protection. Molecules.

